# Advancing neurosurgical education in the age of online learning and global knowledge sharing: impact of Cerebrovascular Q&A webinar series

**DOI:** 10.3389/fsurg.2023.1274954

**Published:** 2023-11-30

**Authors:** Umme Habiba Faisal, Yassine Alami Idrissi, Bipin Chaurasia, Alexis Takasumi, Matias Baldoncini, Akshal Patel, Stephen Monteith, Cameron McDougall, Matias Costa

**Affiliations:** ^1^ Department of Neurological Surgery, Mayo Clinic, Jacksonville, FL, United States; ^2^ Department of Radiology, University of Pittsburgh, Pittsburgh, PA, United States; ^3^ Hillman Cancer Center, University of Pittsburgh Medical Center, Pittsburgh, PA, United States; ^4^ Department of Neurosurgery, Neurosurgery Clinic, Birgunj, Nepal; ^5^ Seattle Science Foundation, Seattle, WA, United States; ^6^ Department of Neurological Surgery, Hospital San Fernando, Buenos Aires, Argentina; ^7^ Cerebrovascular Neurosurgery, Swedish Neuroscience Institute, Swedish Medical Center, Seattle, WA, United States

**Keywords:** cerebrovascular Q&A, virtual education, webinar, neurosurgery, diversity

## Abstract

**Background:**

The Seattle Science Foundation created the Cerebrovascular Q&A series as a free web-based tool to educate physicians and physicians-in-training about cerebrovascular and endovascular neurosurgery across geographical boundaries and different levels of training.

**Objective:**

This study aims to assess the educational impact and clinical implications of the Cerebrovascular Q&A webinar series, hosted by the Seattle Science Foundation.

**Methods:**

A digital anonymous, self-administered survey was sent to the live webinar participants. The survey contained questions about the socio-demographic characteristics of the participants, their perception of the content of the webinar series, and its impact on academic and clinical practice. The data collected from the Survey-Monkey platform was exported to Microsoft Excel which was used to perform all statistical analyses. The viewer metrics on Zoom and YouTube were also analyzed to understand trends observed among a diverse global cohort of participants.

**Result:**

A total of 2,057 people hailing from 141 countries had registered for the Cerebrovascular Q&A series. The response rate to the questionnaire was 12.63% (*n* = 260). Respondents hailed from 65 countries, of which the majority were from India (13.46%, *n* = 35) and United States (11.15%, *n* = 29). Most of the participants were male (82.69%, *n* = 215), while only 15.77% (*n* = 41) were female. The maximum number of participants were neurosurgery attendings (36.65%, *n* = 92) followed by neurosurgeons undergoing fellowship training (24.70%, *n* = 62) and students who were currently in residency training (15.54%, *n* = 39). 75.97% (*n* = 196) heard of the Cerebrovascular Q&A series through the emails from Seattle Science Foundation. 21.5% (*n* = 56) learned about the webinar series through social media. 75% of participants reported that the webinar content was advanced and comprehensive, and the selection of speakers was relevant. 63.08% (*n* = 164) found the webinars sparked innovative research ideas. Additionally, 55% (*n* = 143) reported changes in their clinical practice based on the acquired knowledge.

**Conclusion:**

The findings from this study reveal that webinar-based medical education in cerebrovascular neurosurgery is highly effective and influential. Web-based platforms and social media present a potent strategy to overcome barriers, emphasizing the need for targeted efforts to engage more women in medicine and neurosurgery recruitment.

## Introduction

Online education, especially in the aftermath of the COVID-19 pandemic, has gained significance as a rapid knowledge dissemination tool. The global crisis acted as a pivotal moment in the realm of digital innovation, pushing educational institutions worldwide to adapt to remote learning strategies to comply with social distancing norms. Consequently, the shift from “in-person” to online education became inevitable (1–3). This had profound implications on medical education, leading to deficiencies in both formal curricula and extracurricular learning opportunities. Neurosurgical residents and medical students encountered unique obstacles that hindered networking, research productivity, and clinical instruction (4). Consequently, in this era global knowledge sharing facilitated by social media and web-based seminars, popularly known as “webinars”, have emerged as vital components of global education. These webinars offer unparalleled benefits such as easy accessibility, convenience, and flexibility. Furthermore, other innovative methods of knowledge dissemination, such as podcasts and intraoperative video recordings, have gained popularity and become mainstream to bridge the knowledge gap (5).

The Seattle Science Foundation a physician-driven non-profit organization is committed to improving patient care through education, research, innovation, and technology. It holds accreditation from the Accreditation Council for Continuing Medical Education (ACCME) to provide continuing medical education for physicians (6). The Cerebrovascular Q&A series, conceptualized by the Seattle Science Foundation, is an open-access, free, web-based educational tool. It is designed to impart knowledge from leaders in the field of cerebrovascular and endovascular neurosurgery to an audience across geographical barriers and various levels of training. This is achieved through a series of webinars that can be viewed in real-time as well as retrospectively (7). This article explores the educational impact and clinical implications of the Cerebrovascular Q&A series hosted by the Seattle Science Foundation in the field of cerebrovascular/endovascular neurosurgery, shedding light on the potential application of online webinars as a digital educational tool.

## Methods

This project was exempted from IRB approval due to its non-clinical nature and the absence of patient-related data incorporated into the study.

### Webinar series

The Seattle Science Foundation hosted a live webinar series called Cerebrovascular Q&A series. The series was conceptualized by MC and CM. Course enrollment commenced in the first week of September 2021 and has remained open until the time of preparation of this manuscript. The sessions were conducted live every two weeks over a video conferencing platform (Zoom Video Communications Inc., San Jose, California, USA) and simultaneously streamed on YouTube. The series was made available for asynchronous viewing on the Seattle Science Foundation YouTube channel (7). Participants had the flexibility to register for the course and watch the live events on Zoom, attend lessons directly without course registration through live streaming on YouTube, or view recorded versions later on YouTube. As of the time of analysis, the webinar series ran from October 2021 to July 2023, comprising a total of 40 sessions. A chronological list of episodes is provided in the Supplementary Material.

### Survey

A digital anonymous, self-administered survey was emailed to all regular course participants (Zoom subscribers) since they were the only ones who provided their contact details. Survey details can be found in the Supplementary Material. The survey collected attendees' socio-demographic characteristics including date of birth, country of residence, and current employment role/level of training. Participants' perception of the webinar series and its impact on academic and clinical practice was assessed through questions answered on a 5-point Likert scale with the options ranging from “disagree”, to “neither agree nor disagree”, to “agree”. Data analytics from Zoom and YouTube platforms included the number of live viewers per session, recorded views, and total views.

The collected data was exported from the SurveyMonkey platform to Microsoft Excel (Microsoft Corp., Redmond, Washington, USA) for all statistical analyses. Numerical variables were analyzed for mean values along with standard deviations. Additionally, an analysis of viewer feedback was conducted, including questions about the usefulness of the webinars for theoretical understanding and clinical practice.

## Results

**Figure 1:**
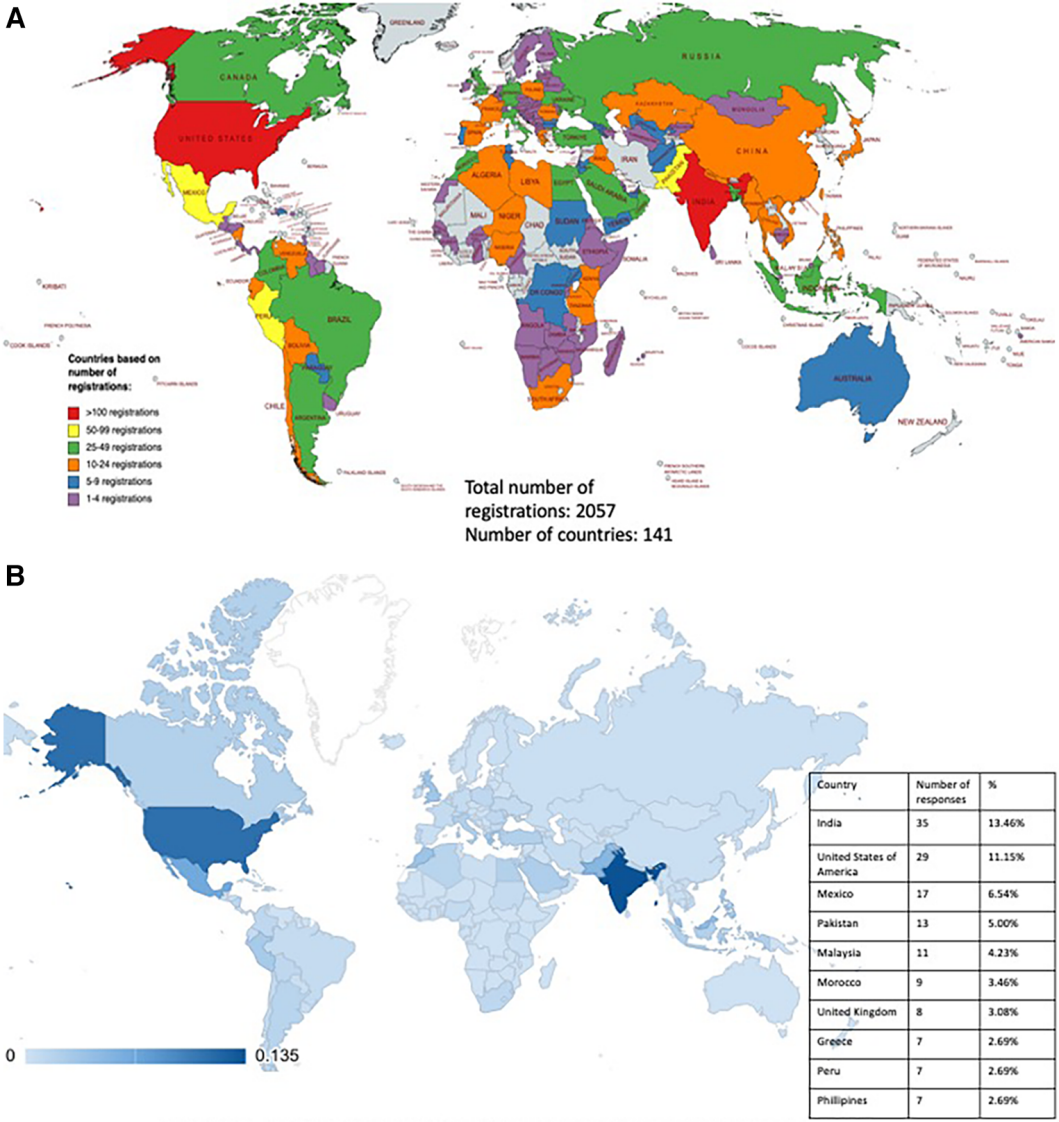
(**A**) Country demographics of cerebrovascular Q&A registrants. Total number of registrations: 2,057. Number of countries: 141. (**B**) Country demographics of cerebrovascular Q&A Survey respondents (*N* = 260) with insert showing the top 10 countries with the most responses.

**Figure 2:**
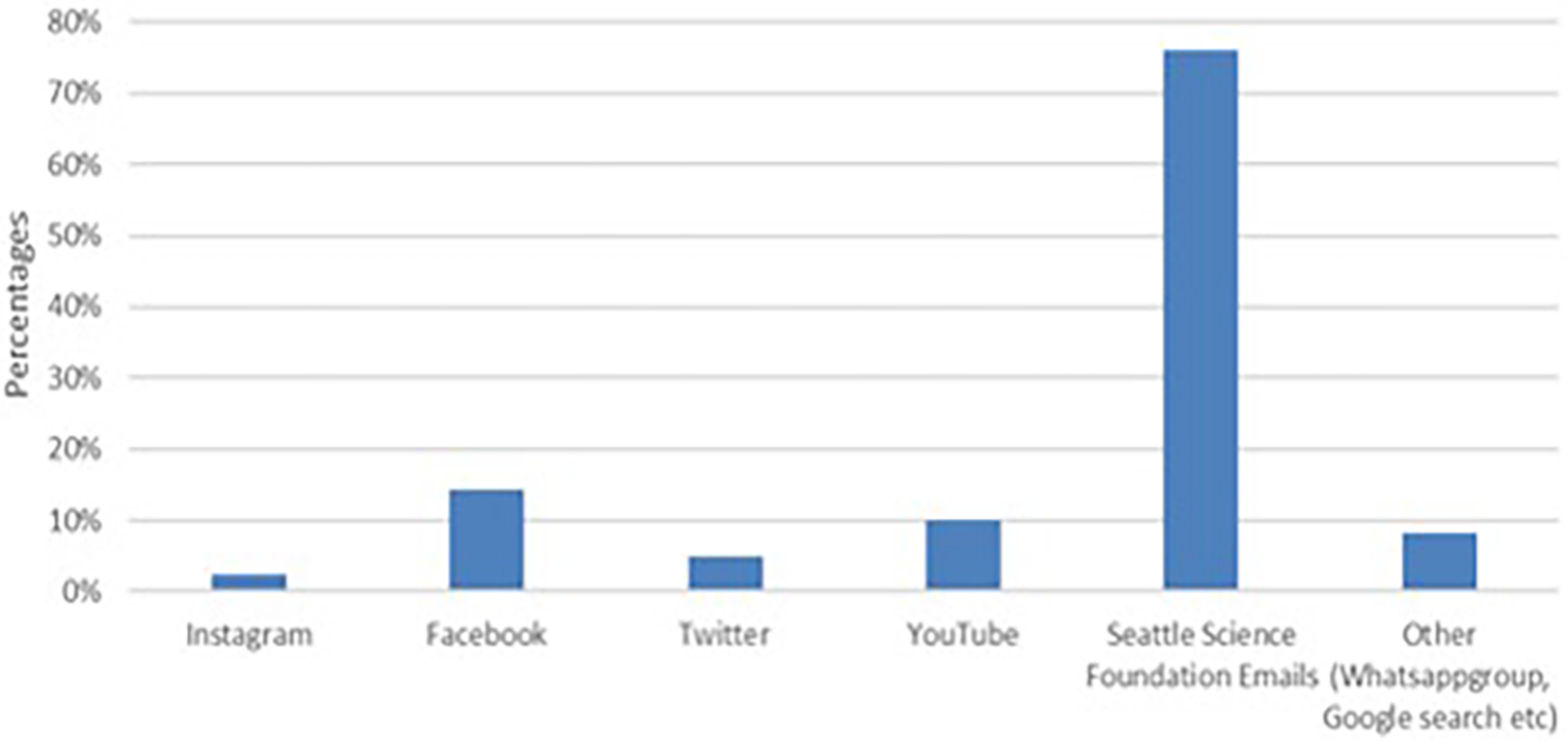
Social media platforms through which participants heard of the cerebrovascular Q&A series.

At the time of preparation of the manuscript (30th July 2023), a total of 2,057 people had registered for the Cerebrovascular Q&A series, representing 141 countries (Figure 1A). The survey was e-mailed to 2,057 registrants, garnering a response rate of 12.63% (*n* = 260). Respondents from 65 countries participated, with the highest proportion from India (13.46%, *n* = 35), closely followed by the United States (11.15%, *n* = 29). The remaining respondents hailed from the rest of the 63 different nations, reflecting a diverse geographic distribution (Figure 1B). The majority of participants identified as male (82.69%, *n* = 215), whereas only 15.77% (*n* = 41) identified as female. The mean age of the participants was 43.38 ± 3.53 years. Most participants learned about the Cerebrovascular Q&A series through e-mails from Seattle Science Foundation (75.97%, *n* = 196), while 21.5% (*n* = 56) of discovered it through Social Media platforms such as X, Facebook, and Instagram (Figure 2).

Upon stratifying participants by their self-reported levels of training, 251 participants answered the question (*n* = 260) with 9 respondents skipping the question altogether. Out of 85 participants who initially chose as “Other”, 64 were reclassified under the provided answer choices. The majority of participants were neurosurgery attendings (36.65%, *n* = 92), followed by neurosurgeons in subspeciality fellowships (24.70%, *n* = 62) and physicians in residency training (15.54%, *n* = 39). A detailed breakdown of survey participants can be found in Table 1.

**Table 1:** Demographic characteristics of survey respondents (*N* = 260).

Characteristics		*n*	Percentage (%)
No. of respondents		260	
Age, years	Mean	43.38 ± 3.53	
Gender (*n*, %)	Male	215	82.69
	Female	41	15.77
	Prefer not to say	4	1.54
Level of education/training	College	5	1.99
	Medical school	8	3.19
	Medical residency	39	15.54
	Fellowship: radiology	5	1.99
	Fellowship: neurology	3	1.20
	Fellowship: neurosurgery	62	24.70
	Attending: radiology	13	5.18
	Attending: neurology	3	1.20
	Attending: neurosurgery	92	36.65
	Other	21	8.37

At the time of preparation of this manuscript, a total of 40 lectures were posted to the YouTube channel, accumulating 62,356 views and averaging of 1,559 views per session. Video live streaming on Zoom was the most common means of viewing the webinar series (81.78%, *n* = 211), followed closely by YouTube (54.43%, *n* = 141). More viewers watched the recorded videos on YouTube (35.27%, *n* = 91) than those who joined the YouTube live stream (19.38%, *n* = 50). Zoom viewer metrics indicated an average of 68 unique views per session (range = 37–103) and 43 concurrent views per session (range = 25–65). (A chronological list of webinar episodes can be found in Supplementary Material).

Regarding the content, 75.77% (*n* = 197) of the participants found the webinar series to be advanced and comprehensive. Similarly, (75%, *n* = 195) reported that the selection of speakers was relevant while 14.23% (*n* = 37) reported that the speakers would rarely be encountered giving lectures in the participant's home country. Another 9.62% (*n* = 25) reported that the speakers in the session were rarely accessible beyond the purview of the webinar series.

**Figure 3:**
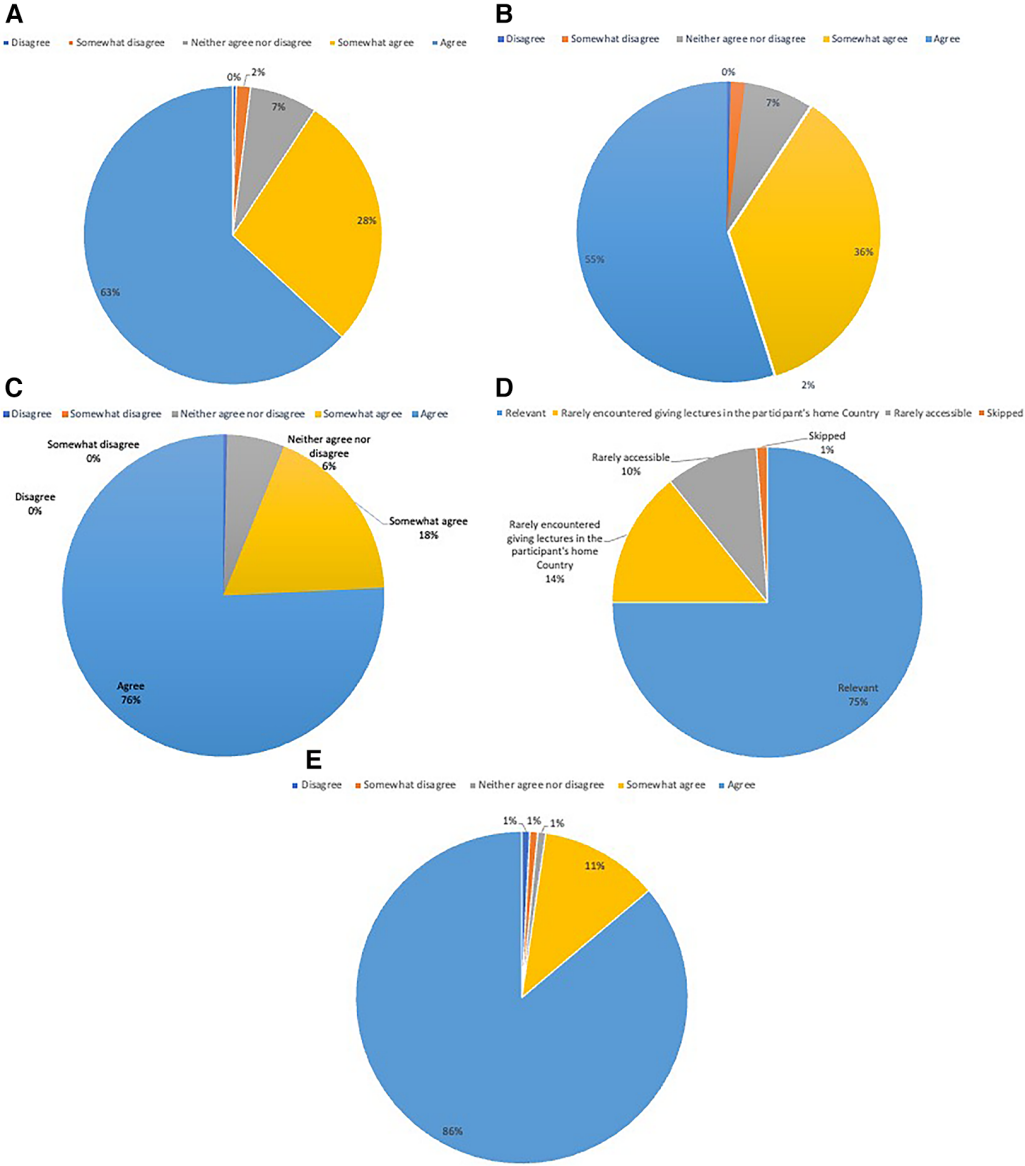
Participants perception of the cerebrovascular Q&A series.

An analysis of viewer feedback revealed that 63.08% (*n* = 164) of participants agreed that the webinar series inspired thought-provoking ideas for research projects, while more than half, i.e., 55% (*n* = 143) of the participants, reported that the knowledge gained through these sessions helped them change at least one aspect of their clinical practice. 86.15% (*n* = 224) expressed eagerness to participate in future sessions (Figures 3A–E).

## Discussion

### Bridging gaps with webinars: accessible, flexible, and convenient

The increase in accessibility and convenience of webinars has led to a remarkable surge in attendance, especially since the onset of the COVID-19 pandemic. This trend is corroborated by a cross-sectional study conducted by Ismail et al. (8), which surveyed 326 physicians with 195 (59.8%) reporting attending more webinars since the pandemic.

Webinars offer substantial advantages over in-person events, providing convenient online access through platforms like Zoom and YouTube, as seen in the Cerebrovascular Q&A series. The series was developed as an accessible web-based educational tool, aimed at disseminating knowledge from vascular neurosurgery experts to a diverse audience, transcending geographical barriers and varying levels of medical training. This approach became particularly vital during the pandemic that disrupted traditional in-person and practical medical education. This accessibility liberated attendees from the constraints of costly and impractical physical attendance, especially considering geographical limitations, as evidenced by the widespread registration (*n* = 2,057) from 141 countries. Although participation in our exit survey was only 12.36%, the responses indicate that we were able to reach a broad audience across 65 nations. This widespread attendance can be attributed to the user-friendly and accessible format of the Cerebrovascular Q&A series.

While technical issues such as internet connectivity or software malfunctions can affect the webinar's quality and disrupt the learning experience, post-live access underscores the flexibility of webinars. Participants can conveniently access content at their own pace, mitigating issues that may arise during live event. For example, the Cerebrovascular Q&A series was recorded and made accessible on the Seattle Science Foundation YouTube channel. This allowed viewers to watch asynchronously, providing unrestricted access to valuable content. This flexibility is evident in viewer metrics with 81.78% (*n* = 211) watching the live stream on Zoom, closely followed by YouTube (54.43%, *n* = 141). Additionally, 35.27% (*n* = 91) viewed the recorded videos on YouTube. This is further corroborated by the analysis of viewer metrics on Zoom and YouTube. The data showed that, on average, each Zoom session attracted around 68 unique views (range between 37 and 103), and approximately 43 concurrent views per session (range = 25–65). These numbers indicate substantial engagement and interest during live broadcasts. Moreover, the YouTube featuring 40 lectures garnered a total of 62,356 views, with an average of 1,559 views per session. These highlights continue interest even after live broadcasts, as participants and viewers continue to access and engage with the content asynchronously.

The advantages of this flexibility were also observed in other webinars, such as the one organized by the Department of Neurosurgery at Lenox Hill Hospital, titled “BRAINterns”. It employed both synchronous and asynchronous methods, including didactic seminars, multimedia presentations, and “live” operative experiences. This approach effectively replaced lost opportunities and garnered significant satisfaction from participants. In fact, 93% of participants found the BRAINterns experience valuable during the pandemic, with 86% feeling that it compensated for missed learning opportunities (9).

The Cerebrovascular Q&A series benefitted not only neurosurgical attendings, fellows, and residents but also allied neuro-interventional participants, including fellows and attendings in the fields of neurology and radiology (Table 1). This diverse audience composition highlights the webinar's relevance and appeal, catering to various medical professionals seeking to enhance their expertise in cerebrovascular/endovascular neurosurgery and related disciplines.

Webinars have become a transformative force in the realm of education, by leveraging digital platforms to deliver knowledge conveniently and efficiently, even in challenging circumstances. Their accessibility and adaptability mark a turning point in global education, providing diverse learning opportunities for individuals worldwide.

### Webinars for inclusivity: need to enhance recruitment of women in neurosurgery

Over the last decade, there has been a significant increase in the enrollment of women in U.S. medical schools, reaching an unprecedented majority of 50.7% in 2017 (10). Despite this progress, the representation of women neurosurgeons (WNS) in residency training has remained notably low. The AAMC's 2022 Physician Specialty Data Report (11) which reported the numbers of physicians, residents, and fellows among the 48 largest specialties in 2021 in the United States, found that the representation of women in neurosurgery is abysmally low comprising a mere 9.6% of the resident workforce. Women orthopedic surgeons face a similar challenge, with their representation standing at a mere 5.9% during the same period. This gender disparity extends to leadership positions within major U.S. national neurosurgery organizations, a trend that is also observed on a global scale (10–13).

As per the survey conducted by the European Women in Neurosurgery (E-WIN) Project, the overall proportion of women neurosurgeons (WNS) in Europe stands at 12%, with 1,565 out of 12,985 respondents being women (14). Italy reported the highest overall proportion of women neurosurgeons (WNS) at 36%, while Kosovo and Cyprus had no WNS. In Japan, WNS account for 1.3% of board-certified neurosurgeons. In developing countries, the situation is considerably bleaker, with some nations having only a handful of neurosurgeons to cater to millions of people (15). Our survey findings further highlight this gender gap, indicating that out of the 260 participants, only 15.77% (*n* = 41) were females, while males had an overwhelmingly greater representation of 82.6% (*n* = 215) (Table 1).

The global gender gap in neurosurgery is widely recognized, and although various theories about its causes abound, providing definitive evidence remains elusive. However, the positive impact of closing the gender and diversity gaps has already been well-documented in various industries (16). Consequently, it is crucial to foster a culture change in medical institutions, encouraging diversity and inclusion by motivating more women to join the workforce, including in the field of neurosurgery. Attracting more women to neurosurgery can be achieved through diverse strategies such as mentoring, teaching leadership, and negotiation skills, and implementing job sharing or dual training tracks (11).

Research indicated that early preclinical exposure significantly inspires students to pursue a career in neurosurgery (17). As discussed earlier, the accessibility and convenience of webinars in providing free neurosurgical exposure to a predominantly preclinical student audience can yield favorable long-term implications for diversifying and expanding the pool of potential neurosurgery applicants.

### Opportunity to interact, engage and ask questions in real-time

Live webinars have a distinct advantage of over offline educational content, particularly in the realm of interactivity. While in-person interactions can sometimes be more effective in conveying certain ideas or questions, webinars offer unique benefits through both oral interactions and chat-based exchanges (18). This proves particularly advantageous in global conferences where attendees hail from diverse backgrounds, and different accents might pose challenges to mutual understanding (19). An example of this was observed during a live webinar hosted by the International Association of Oral and Maxillofacial Surgeons (IAOMS) on orthognathic surgery where participants actively engaged in a live, interactive chat with the presenter. This interactive approach allowed everyone to follow the ongoing discussions and greatly enhanced the overall learning experience (18).

While online webinars offer high interactivity, they may lack the face-to-face interaction found in in-person conferences, limiting networking opportunities and relationship-building with colleagues. However, webinars provide a valuable chat feature, acting as a lifeline for shy individuals who might hesitate to participate verbally, allowing them to confidently ask questions and gain additional knowledge and insights, which might have been withheld in an in-person setting. This digital exchange fosters active engagement, breaking down communication barriers, and enriching the learning experience.

Similarly, the Cerebrovascular Q&A series provided the participants the opportunity to interact with the speakers. This is evidenced by the fact that 9.62% (*n* = 25) participants stated that the speakers in the session were rarely accessible beyond the scope of the webinar series.

### Opportunity for mentorship and networking

The National Institutes of Health (NIH) recognizes the importance of recruiting and training scholars from underrepresented minority (URM) backgrounds (20). This approach is seen as essential to foster inclusive leadership in science and innovation, as well as to make strides in reducing health disparities for underserved populations (21, 22). Scholars originating from URM backgrounds and/or Low- and Middle-Income Countries (LMICs) encounter numerous obstacles in advancing their careers. These challenges encompass increased financial burdens, social and professional isolation, as well as biases associated with race or ethnicity (21–24). Mentorship can help overcome these barriers and mentors can play a pivotal role in guiding their mentees through the intricacies of institutional culture, assisting them in devising strategies to overcome barriers related to race, and charting well-defined career paths to attain their desired objectives (25–27). Webinars, such as Cerebrovascular Q&A, offer an excellent avenue for networking and the potential for mentorship to emerge by connecting students and researchers with subject experts. The survey results provide evidence in this regard, with 14.23% (*n* = 37) of participants reporting that the speakers were rarely encountered giving lectures in their home country. Additionally, 9.62% (*n* = 25) stated that the speakers in the session were rarely accessible beyond the scope of the webinar series.

The inherent accessibility of webinars from anywhere in the world enables individuals from diverse regions to connect and establish professional relationships within their respective fields. Such global interactions become especially valuable, as they facilitate real-time engagement, allowing participants to exchange ideas and connect on shared interests (28). These interactions create a sense of camaraderie and shared learning, often laying the foundations for deeper networking and mentorship opportunities to develop.

Undoubtedly, many medical procedures and techniques necessitate hands-on training, posing challenges in replicating such experiences in an online webinar format. Nevertheless, webinars hold significant value in providing high-quality introduction to these procedures and the potential to connect with mentors virtually, paving the way for valuable real-life mentoring experiences.

### Limitations

This study has several limitations. Firstly, the questionnaire being self-administered, renders the responses susceptible to biases (29–31). Previous studies have shown that respondents tend to often provide positive answers when addressing questions related to satisfaction (32). Additionally, some respondents might have tended to choose the top-listed responses, considering them as high priority, or they might have had a tendency to respond in a certain way, such as always selecting the middle option (31, 33). Furthermore, self-administered surveys typically tend to yield low response rates as shown by the low response rate in our study (34). This low response rate may be attributed to attrition bias; implying that respondents who were less keen on academic pursuits, might have opted out of the survey. Also, there is a possibility that some participants were less willing to express less desirable feedback due to concerns over anonymity (32). Another significant limitation is the absence of data about participants' ethnicity, preventing an analysis of the involvement of minorities and underrepresented groups in our study. Lastly, it is important to note that our study was not designed to find causality. It remains a purely descriptive analysis of the impact of the Cerebrovascular Q&A series on the practice and understanding of neurosurgery.

## Conclusion

The survey results shed light on the positive educational and clinical implications of the Cerebrovascular Q&A series. The findings from this study reveal that webinar-based medical education can be potentially effective, demonstrating their ability to influence a broad audience including historically underrepresented populations.

It can be challenging to assess learning outcomes and evaluate the effectiveness of online webinars compared to in-person conferences. To gain a comprehensive understanding of their true impact, future studies should consider conducting original research comparing the same online seminar with an equivalent in-person event. Such comparative studies hold the key to more insightful and accurate assessments, offering a promising avenue for further exploration into this relatively new phenomenon. Undertaking such research endeavors, will provide valuable insights that will contribute to the continued advancement and optimization of online medical education, particularly in the field of neurosurgery.

## Data Availability

The raw data supporting the conclusions of this article will be made available by the authors, without undue reservation.
