# Supplementary material for: Advancing neurosurgical education in the age of online learning and global knowledge sharing: impact of Cerebrovascular Q&A webinar series
Source: Front Surg. 2023 Nov 30;10:1274954. doi: 10.3389/fsurg.2023.1274954 (PMC10723828; doi:10.3389/fsurg.2023.1274954)
Supplement: Supplementary file 1 [file Datasheet1.pdf]

Table 1 : Chronological list of topics covered in the Cerebrovascular Q&A webinar series, along with the list of speakers.

Sr No	Video : Topic & Speaker	Release date
1	Cerebrovascular Q&A: Revascularization for Moyamoya Disease- Bi Xu, M.D.	Oct 29, 2021
2	CV Q&A- The Art of Flow Diversion- Pedro Lylyk, M.D.	Dec 11, 2021
3	Dural AVF's With PIAL Supply - Timo Krings, MD	Jan 12, 2022
4	Skull Base Armamentarium for the Cerebrovascular Neurosurgeon - Vinko Dolenc, M.D.	Nov 12, 2021
5	ECA Dangerous Anastomosis: Key Anatomy and Clinical Cases	Oct 14, 2021
6	Anerysms, Surgical & Clipology - Juha Hernesniemi	Oct 1, 2021
7	VR/AR in Cerebrovascular Neurosurgery - Walter Jean, MD	Jan 26, 2022
8	WEB Aneurysm Treatments - Laurent Pierot, MD	Feb 10, 2022
9	eShunt: The Next Revolution in Hydrocephalus Treatment? - Adel Malek, M.D.	Feb 23, 2022
10	Vessel Wall Imaging in Cerebrovascular Neurosurgery – Mahmud Mossa-Basha, M.D.	Mar 10, 2022
11	From Anatomy to Pathology: The Power of Imaging – Eytan Raz, M.D.	Mar 23, 2022
12	Super Selective Transvenous dAVF Embolization – Maksim Shapiro, M.D.	Apr 13, 2022
13	Glymphatic System: Implications for Cerebrovascular Neurosurgery – Iype Cherian, M.D.	Apr 27, 2022
14	CSF-Venous Fistulas: A New Endovascular Frontier - Waleed Brijinkji, M.D.	May 11, 2022
15	Novel Insights in Imaging and Treatment of Brain AVM's – Timo Krings, M.D.	May 25, 2022
16	WEB Aneurysm Treatment: Results of Prospective Studies - Laurent Pierot, MD	Jun 8, 2022
17	Epidural Vascular Malformations of the Spine - Giuseppe Lanzino, M.D	Jun 22, 2022
18	Functional Vascular Anatomy of The Spine & Spinal Cord – Masaki Komiyama, M.D.	Jul 13, 2022
19	Revascularization for Intracranial Atherosclerotic Steno-Occlusive Disease - Nestor Gonzalez, MD	Jul 27, 2022
20	Perfusion Imaging in Cerebrovascular Ischemia – Jeremy Heit, M.D., Ph.D	Aug 24, 2022
21	When Patients Comes First: Choosing Access Site for the Best Thrombectomy Set Up– Elad Levy, M.D	Sep 14, 2022
22	Robotics in Neurovascular Surgery – Stavropoula Tjoumakaris, M.D	Sep 29, 2022
23	Super-Subspecialization in Neurosurgery: The Vascular Paradigm - Pascal Jabbour, M.D.	Oct 12, 2022
24	Treatment of Giant Aneurysms: State of the Art - Ricardo Hanel, M.D., Ph.D.	Oct 26, 2022
25	Curiosity, A Calling to Research – J. Mocco, M.D.	Nov 9, 2022

26	Tandem Occlusions: Current Evidence and Next Steps - Santiago Ortega-Gutierrez, MD	Nov 23, 2022
27	Decision Making in Surgical Treatment of Intracranial Aneurysms in the Endovascular Era	Dec 14, 2022
28	Surgery to Endovascular Therapy, New Hybrid Approaches: Carotid Cavernous Fistulas - Peter Kan, M.D.	Jan 11, 2023
29	Venous Stenting For IIH And Pulsatile Tinnitus - Vitor Pereira, M.D	Jan 25, 2023
30	Remote Robotic Neuro-Intervention: In Vitro and Computational Simulations - Satoshi Tateshima, M.D	Feb 8, 2023
31	A Lifetime Into Cavernomas: Lessons Learned - Issam Awad, M.D., MSc, FACS, MA	Feb 22, 2023
32	Curative Embolization of Cerebral AVMs - Dr. med René Chapot	Mar 8, 2023
33	High Resolution Intravascular Imaging of The Cerebrovasculature - Matthew Gounis, Ph.D	March 22, 2023
34	The Future of Cerebrovascular Surgery - Dr Gary Steinberg	April 12, 2023
35	Flow diversion and Beyond. Ajay Wakhloo	May 10, 2023
36	Endovascular Management of Chronic Subdural Hemorrhage - David Fiorella, MD	May 24, 2023
37	Endovascular Management of Acute Stroke – Santiago Ortega-Gutierrez, M.D	June 14, 2023
38	Biplane Angiography into the Intraoperative Setting - Jan-Karl Burkhardt, MD	June 28, 2023
39	Endovascular therapy for stroke. Current status and future directions - Tudor Jovin, MD	July 12, 2023
40	Neurosurgical Artificial Intelligence: Opportunities and Challenges – Daniel Donoho, MD	July 26, 2023

Survey Questions/Questionnaire:

1. What country do you live in?
2. If living in the United States, which state do you reside in?
3. In what year were you born?
4. What is your gender?
   - ○ Male
   - ○ Female
   - ○ Prefer not to say

5. What phase of education/training/practice are you currently in?
   - ○ College
   - ○ Medical school
   - ○ Medical residency
   - ○ Fellowship: Radiology
   - ○ Fellowship: Neurology
   - ○ Fellowship: Neurosurgery
   - ○ Attending: neurointerventional radiology
   - ○ Attending: Neurointerventional neurology
   - ○ Attending: neurointerventional neurosurgery
   - ○ Other (please specify)

6. Where do you usually watch the Q&A webinars?
   - ○ Zoom live streaming
   - ○ YouTube live streaming
   - ○ YouTube recorded sessions
   - ○ Other
   - ○ Other (please specify)

7. How did you hear about this webinar?
   - ○ Instagram
   - ○ Facebook
   - ○ Twitter
   - ○ YouTube
   - ○ Seattle Science Foundation Emails
   - ○ Other (Whatsappgroup, Google search etc)

8. The sessions have allowed me to come up with thought-provoking ideas which can be considered for research projects.
   - ○ Disagree
   - ○ Somewhat disagree
   - ○ Neither agree nor disagree
   - ○ Somewhat agree
   - ○ Agree

9. The sessions have allowed me to change at least one aspect of my clinical practice.
   - ○ Disagree
   - ○ Somewhat disagree
   - ○ Neither agree nor disagree
   - ○ Somewhat agree
   - ○ Agree

10. The content of the sessions is advanced and comprehensive
    - ○ Disagree
    - ○ Somewhat disagree
    - ○ Neither agree nor disagree
    - ○ Somewhat agree
    - ○ Agree
11. The speakers invited are:
    - ○ Relevant
    - ○ Rarely encountered giving lectures in the participant's home Country
    - ○ Rarely accessible

12. I look forward to continuing to join future sessions in the series.
    - ○ Disagree
    - ○ Somewhat disagree
    - ○ Neither agree nor disagree
    - ○ Somewhat agree
    - ○ Agree
